# Radiologic imaging biomarkers in triple-negative breast cancer: a literature review about the role of artificial intelligence and the way forward

**DOI:** 10.1093/bjrai/ubae016

**Published:** 2024-11-13

**Authors:** Kanika Bhalla, Qi Xiao, José Marcio Luna, Emily Podany, Tabassum Ahmad, Foluso O Ademuyiwa, Andrew Davis, Debbie Lee Bennett, Aimilia Gastounioti

**Affiliations:** Breast Image Computing Lab, Washington University School of Medicine in St. Louis, St. Louis, MO 63110, United States; Mallinckrodt Institute of Radiology, Washington University School of Medicine in St. Louis, St. Louis, MO 63110, United States; Mallinckrodt Institute of Radiology, Washington University School of Medicine in St. Louis, St. Louis, MO 63110, United States; Mallinckrodt Institute of Radiology, Washington University School of Medicine in St. Louis, St. Louis, MO 63110, United States; Alvin J. Siteman Cancer Center, Washington University School of Medicine in St. Louis, St. Louis, MO 63110, United States; Division of Hematology, Department of Medicine, Washington University School of Medicine in St. Louis, St. Louis, MO 63110, United States; Division of Oncology, Department of Medicine, Washington University School of Medicine in St. Louis, St. Louis, MO 63110, United States; Mallinckrodt Institute of Radiology, Washington University School of Medicine in St. Louis, St. Louis, MO 63110, United States; Alvin J. Siteman Cancer Center, Washington University School of Medicine in St. Louis, St. Louis, MO 63110, United States; Division of Oncology, Department of Medicine, Washington University School of Medicine in St. Louis, St. Louis, MO 63110, United States; Alvin J. Siteman Cancer Center, Washington University School of Medicine in St. Louis, St. Louis, MO 63110, United States; Division of Oncology, Department of Medicine, Washington University School of Medicine in St. Louis, St. Louis, MO 63110, United States; Mallinckrodt Institute of Radiology, Washington University School of Medicine in St. Louis, St. Louis, MO 63110, United States; Alvin J. Siteman Cancer Center, Washington University School of Medicine in St. Louis, St. Louis, MO 63110, United States; Breast Image Computing Lab, Washington University School of Medicine in St. Louis, St. Louis, MO 63110, United States; Mallinckrodt Institute of Radiology, Washington University School of Medicine in St. Louis, St. Louis, MO 63110, United States; Alvin J. Siteman Cancer Center, Washington University School of Medicine in St. Louis, St. Louis, MO 63110, United States

**Keywords:** radiomics, artificial intelligence, deep learning, machine learning, triple-negative breast cancer, breast cancer, predictive biomarkers, prognostic biomarkers

## Abstract

Breast cancer is one of the most common and deadly cancers in women. Triple-negative breast cancer (TNBC) accounts for approximately 10%-15% of breast cancer diagnoses and is an aggressive molecular breast cancer subtype associated with important challenges in its diagnosis, treatment, and prognostication. This poses an urgent need for developing more effective and personalized imaging biomarkers for TNBC. Towards this direction, artificial intelligence (AI) for radiologic imaging holds a prominent role, leveraging unique advantages of radiologic breast images, being used routinely for TNBC diagnosis, staging, and treatment planning, and offering high-resolution whole-tumour visualization, combined with the immense potential of AI to elucidate anatomical and functional properties of tumours that may not be easily perceived by the human eye. In this review, we synthesize the current state-of-the-art radiologic imaging applications of AI in assisting TNBC diagnosis, treatment, and prognostication. Our goal is to provide a comprehensive overview of radiomic and deep learning-based AI developments and their impact on advancing TNBC management over the last decade (2013-2024). For completeness of the review, we start with a brief introduction of AI, radiomics, and deep learning. Next, we focus on clinically relevant AI-based diagnostic, predictive, and prognostic models for radiologic breast images evaluated in TNBC. We conclude with opportunities and future directions for AI towards advancing diagnosis, treatment response predictions, and prognostic evaluations for TNBC.

## Introduction 

Breast cancer is a common disease affecting almost 2 million women globally each year.^1^ The rates of breast cancer have been increasing steadily over the past several decades and despite advances in treatment, breast cancer still accounts for 15% of estimated cancer deaths in women.^2^ Breast cancer is comprised of 4 main molecular subtypes based on the expression of oestrogen receptor (ER), progesterone receptor (PR), and human epidermal growth factor receptor 2 (HER2) by the breast cancer cells: luminal A, luminal B, HER2-enriched (HER2+), and triple-negative/basal-like.^3^ These subtypes differ in their imaging appearance, disease course, response to treatment, and survival rates. The group of tumours that express neither hormone receptors nor HER2 are known as “triple-negative breast cancer (TNBC)” or basal-like breast cancers.

TNBC accounts for approximately 10%-15% of breast cancer diagnoses and is more common in people with a BRCA1 mutation, younger women, and Black women.^4^ The diagnosis and management of TNBC represent a challenge. TNBC can be difficult to diagnose visually in radiologic images, due to its imaging features which can overlap with benign entities, and may also vary by race.^5^ Moreover, TNBC is an aggressive form of breast cancer. Compared to other molecular breast cancer subtypes, TNBC tends to be higher grade and larger in size at time of diagnosis with high rate of recurrence and distant metastases. The 5-year relative survival rate for women with TNBC is 77.1%, compared with 94.4% for women with HR+/HER2− cancers.^6^ Because TNBC lacks hormone receptors and HER2 for targeted therapies, women with early-stage TNBC receive either neoadjuvant or adjuvant chemotherapy with the inclusion of pembrolizumab for patients with stage II and III TNBC; however, systemic therapy is not fully effective for all patients and a subset of patients will develop metastatic disease.^7^

These clinical challenges associated with TNBC have posed the urgent need for developing more effective and personalized imaging biomarkers to guide TNBC diagnosis, treatment, and prognostication. Radiologic imaging is used routinely for TNBC diagnosis, staging, and treatment planning.^8^ Moreover, with its potential for high-resolution 3D tissue visualization, radiologic imaging provides a unique means for capturing vital aspects of whole-tumour characteristics in vivo.^9^ However, radiologic images offer insights into anatomical and functional properties of tumours that may not be easily perceived by the human eye and thereby may be underexploited. This offers unique opportunities for computerized radiologic imaging tools, which can capture fine, often-imperceptible-to-humans details of radiologic imaging patterns that reflect the genetic and molecular characteristics of the tissues. In recent years, the computational medical imaging community has taken notice of an artificial intelligence (AI) revolution driven by high-throughput features of image patterns (called “radiomics”) and deep learning, which have brought AI to the mainstream in medical image computing. AI has not only expanded the utility of radiologic imaging in predictive and prognostic models but has also pervaded breast cancer screening as one of the most promising computerized imaging tools.

In this review, we synthesize the current state-of-the-art radiologic imaging applications of AI in assisting TNBC diagnosis, treatment, and prognostication. For a more complete view of AI updates in TNBC management, we refer the reader to excellent recent review papers and perspective articles on AI applications on multi-omics, including radiologic, histopathology, gene expression, and living cell imaging data.^10–12^ This review focuses on radiologic imaging and provides a comprehensive overview of radiomic and deep learning-based AI developments and their impact on advancing TNBC management over the last decade. For completeness of the review, we start with a brief introduction of AI, radiomics, and deep learning. Next, we focus on clinically relevant AI-based diagnostic, predictive and prognostic models evaluated in TNBC. We conclude with opportunities and future directions for AI towards advancing diagnosis, treatment response predictions, and prognostic evaluations for TNBC.

## The advent of AI in breast image computing

AI is an emerging technology designed for modelling intelligent behaviours under minimal human interventions. In the past few years, AI techniques have been applied to almost every facet of oncology, from basic research to drug development and clinical care.^13^ Computerized AI tools in breast imaging aim to capture fine details of breast imaging patterns that reflect the genetic and molecular characteristics of the tissues and are often-imperceptible-to-humans. These computerized AI tools include high-throughput features of image patterns (called “radiomics”)^14^ fed into traditional machine learning (ML) models (eg, random forests and support vector machines [SVMs]), as well as deep learning models, the latest revolution in AI, that possess the unique advantage of ingesting images directly without explicit feature conversion.

**Figure 1.**
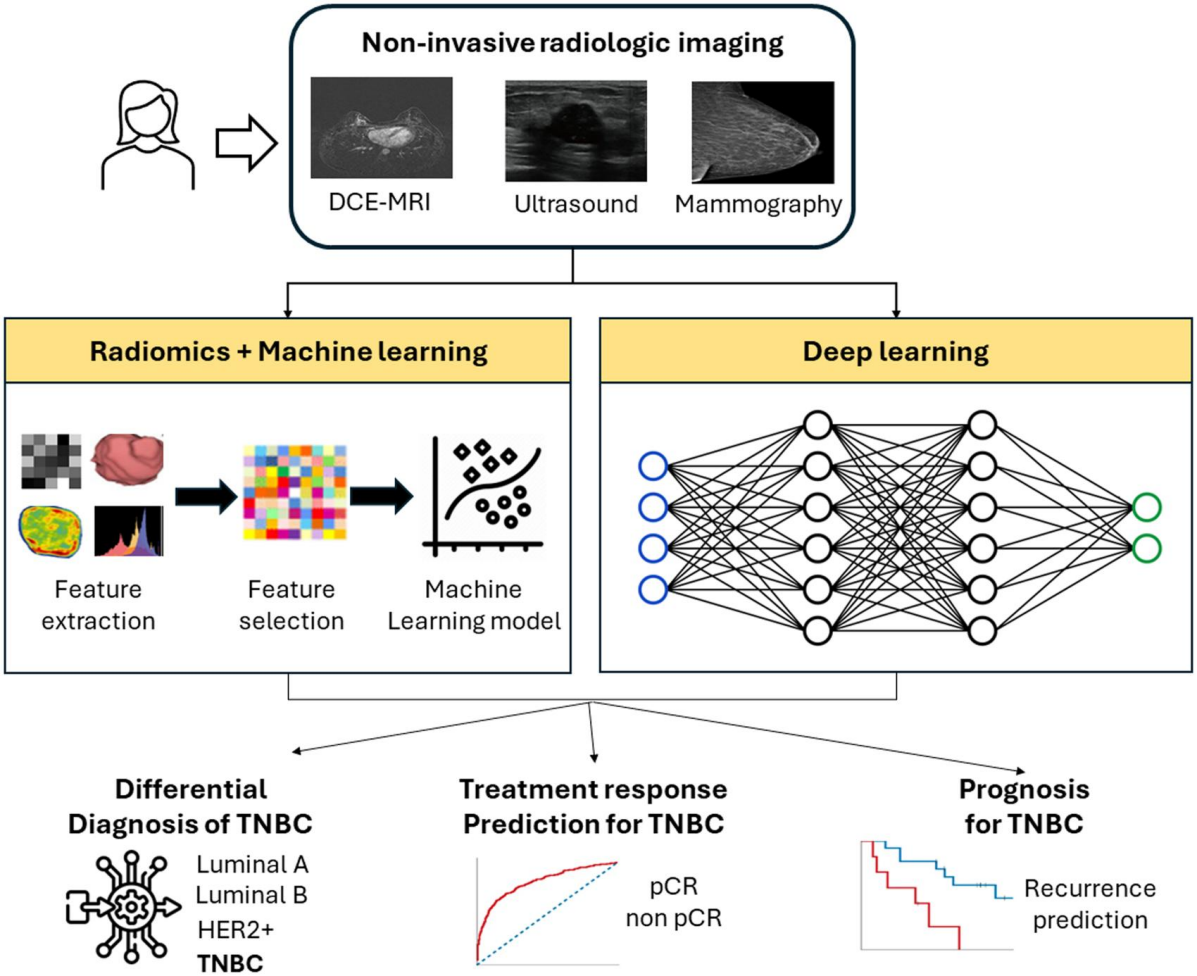
Overview of radiologic imaging applications of AI in assisting TNBC diagnosis, treatment, and prognostication. Abbreviations: AI = artificial intelligence; TNBC = triple-negative breast cancer.

The process of developing a radiomic AI model of tumour phenotypes includes image acquisition, tumour segmentation, feature extraction, feature selection, and model building. For example, if we aim to develop a radiomic AI model in a supervised fashion to predict a clinical outcome using pre-treatment breast images, we draw the regions for the tumours on breast images using automated or semi-automated software, extract hundreds or thousands of quantitative radiomic features using dedicated software, select several features, and then develop a prediction model associating the selected features with the clinical outcome. Deep learning models, on the other hand, can investigate the relationship between input data (eg, pre-treatment breast imaging data) and the clinical outcome from the input data themselves^15^ (Figure 1). Therefore, deep learning does not require the intermediate feature extraction or engineering process, which is laborious, limits knowledge discovery to a pre-defined set of features, and often challenges the model’s reproducibility, especially when in-house software is used. In terms of resources, due to the complexity of their architectures, deep learning models need substantially more training data compared to radiomic AI models. However, their ability to learn directly from the data has demonstrated unprecedented performance in various breast imaging applications, including studies on breast cancer risk assessment,^16^ breast cancer detection^17^ and the differentiation of benign versus malignant lesions.^18^ Beyond supervised AI models, unsupervised AI methods for radiomic phenotyping have proven valuable for discovering unknown phenotypic patterns or clusters in breast cancer imaging, thereby complementing supervised AI by uncovering insights beyond labelled outcome data.^19–21^

## AI in clinical applications of TNBC

We used PubMed as the main source to search relevant publications. The following keywords were used in combination with the keyword of “triple-negative breast cancer”: “artificial intelligence,” “machine learning,” “deep learning,” “radiomics,” “texture.” We also repeated our search using the keyword “breast cancer” rather than “triple-negative breast cancer” and identified relevant publications that performed sub-analyses and reported on performance among TNBC patients. To broaden the search, the “related articles” function provided in PubMed was also used, and all articles and citations obtained were reviewed. The references from all the articles identified were also manually examined for further relevant studies. The last search was conducted on September 23, 2024. Studies not considered relevant to the scope of the review were excluded; other exclusion criteria included: study published before 2013; study not published in the English language, full text not available, letter to the editor, and duplicate publication. In the rest of this section, we summarize key methodological details and evaluation results from the 58 research papers identified by the search. We also report on the publication year of each study, as well as its study design, including sample size, data splits, imaging modality, clinical end points, and type of AI used for analysis.

### Differential diagnosis of TNBC

Because of imaging overlap between TNBC and benign masses, TNBC is often difficult to diagnose with visually assessed radiologic images. Mammographically, TNBC typically presents as a mass without associated calcifications,^22^^,^^23^ and with regular shape and circumscribed margins,^24^ features which are typically associated with benign lesions. Ultrasound features of TNBC can be similarly misleading. Although most TNBC cancers do present as irregular hypoechoic masses without circumscribed margins,^22^^,^^23^ approximately two-thirds of TNBC demonstrate parallel orientation and approximately one-third have posterior enhancement,^22^ which again are features more typical of benign masses. Distinguishing MRI features between TNBC and other breast cancer types have also been reviewed, showing that TNBC cancers often present with a larger tumour size, lobulated shape, smooth margins, and rim enhancement, and are more likely to be unifocal compared with other breast cancer subtypes.^25^ Because of the imaging overlap between TNBC and benign masses, better tools are needed to differentiate these entities to ensure timely and accurate diagnosis.

During the last decade, a hypothesis has evolved suggesting that quantitative assessment of breast images via AI methods elucidates imperceivable tumour characteristics associated with molecular breast cancer subtypes. This hypothesis has been supported by several AI studies (Table 1) reporting moderate to high performances (area under the ROC curve [AUC] = 0.65-0.98 and accuracy = 64.3%-94%) on TNBC classification in various breast imaging modalities. Most studies have reported results characterizing TNBC using dynamic contrast-enhanced (DCE)-MRI alone,^26–37^ diffusion-weighted MRI (DWI) alone,^38–40^ or both MRI sequences in combination.^41–43^ More recently, research groups,^44^^,^^45^ have also investigated the potential of multiparametric MRI (MP-MRI), including ultrafast DCE-MRI, magnetic resonance spectroscopy, diffusion kurtosis imaging (DKI), and intravoxel incoherent motion, which collectively offer a rich set of tumour information, such as blood perfusion, water molecule diffusion, and cell density. For instance, utilizing an MP-MRI approach and a sample of 188 breast cancer patients, Huang et al.^44^ showed that radiomic features combined in a ML classifier were able to characterize TNBC with an AUC of 0.86 (95% CI, 0.71-0.96). Moreover, using SHapley additive exPlanations (SHAP)^46^ for interpreting their ML classifier, the authors found that kinetic information captured in the very early post-contrast period via ultrafast DCE-MRI, as well as DKI features were most predictive of TNBC. Similar performance for radiomics extracted from MP-MRI was also reported by Zhang et al.^45^ on a larger cohort of 477 breast cancer patients, while also showing that the performances of different classifiers on MP-MRI (AUC = 0.75-0.90) were significantly better than on individual MRI sequences (AUC = 0.62-0.88). In addition to breast MRI, multiple studies have also investigated the role of AI applied to breast ultrasound imaging for differentiating TNBC from other molecular breast cancer subtypes,^47–56^ while only a few studies have explored AI approaches for TNBC characterization with CT,^57^^,^^58^ and mammographic imaging modalities,^59–65^ such as the traditional digital mammography and the newer synthetic mammography acquired with digital breast tomosynthesis, and contrast-enhanced mammography.

**Table 1.** Overview of prior AI work on differential diagnosis of triple-negative breast cancer (TNBC).

Study (year)	Imaging modality	Patients/Lesions *N*, All (TNBC) Data splits Use of External Test Set	AI approach	TNBC classification performance
Waugh et al. (2015)^26^	DCE-MRI	148 (24)	Radiomics + ML	AUC: 0.77; Acc: 64.3%
		70%/30%		
		PRoRE		
Wang et al. (2015)^27^	DCE-MRI	84 (11)	Radiomics + ML	AUC: 0.88
		90%/10%		
Moon et al. (2015)^47^	US	169 (85)	Radiomics + ML	AUC: 0.97^a^
		LOOCV		
Li et al. (2016)^28^	DCE-MRI	91 (11)	Radiomics + ML	AUC: 0.67
		LOOCV		
Fox et al. (2016)^29^	DCE-MRI	100 (22)^b^	Radiomics + ML	AUC: 0.94
Sutton et al. (2016)^30^	DCE-MRI	178 (48)	Radiomics + ML	Acc: 81.0%
		LOOCV		
Chang et al. (2016)^31^	DCE-MRI	102 (22)	Radiomics + ML	AUC: 0.83; Acc: 77.45%
		LOOCV		
Fan et al. (2017)^37^	DCE-MRI	96 (20)	Radiomics + ML	AUC: 0.80
		60%/40%		
Guo et al. (2018)^48^	US	215 (51)	Radiomics + ML	AUC: 0.76
		65%/35%		
Saha et al. (2018)^32^	DCE-MRI	922 (164)	Radiomics + ML	AUC: 0.65
		50%/50%		
Ma et al. (2019)^59^	DM	331 (29)	Radiomics + ML	AUC: 0.87; Acc: 79.6%
		90%/10%		
Lee et al. (2018)^49^	US	901 (186)	Radiomics + ML	AUC: 0.83-0.87^a^
		50%/50%		
Chamming et al. (2018)^33^	DCE-MRI	85 (18)	Radiomics + ML	AUC: 0.83
		LOOCV		
Wu et al. (2019)^51^	US	140 (23)	Radiomics + ML	AUC: 0.88
		LOOCV		
Xie et al. (2019)^41^	DCE-MRI, DWI	134 (22)	Radiomics + ML	Acc: 91.0%
		80%/20%		
Leithner et al. (2020)^38^	DWI	91 (23)	Radiomics + ML	Acc: 73.4%
		LOOCV		
Ni et al. (2020)^39^	DWI	112 (11)	Radiomics + ML	Acc: 73.0%
		LOOCV		
Feng et al. (2020)^57^	CE-CT	300 (100)	Radiomics + ML	AUC: 0.85
		60%/40%		
Son et al. (2020)^60^	SM	221 (62)	Radiomics + ML	AUC: 0.84
		65%/35%		
La Forgia et al. (2020)^61^	CEM	68 (7)	Radiomics + ML	AUC: 0.77
		LOOCV		
Leithner et al. (2020)^42^	DCE-MRI, DWI	91 (23)	Radiomics + ML	AUC: 0.86
		70%/30%		
Wang et al. (2020)^64^	DM	54 (23)	Radiomics + ML	AUC: 0.84
		70%/30%		
Demircioglu et al. (2020)^34^	DCE-MRI	95 (15)	Radiomics + ML	AUC: 0.73
		80%/20%		
Wang et al. (2021)^40^	DWI	221 (25)	Radiomics + ML	AUC: 0.82; Acc: 83.7%
		75%/25%		
Krajnc et al. (2021)^58^	FDG-PET/CT	170 (11)	Radiomics + ML	AUC: 0.82
		90%/10%		
Jiang et al. (2021)^50^	US	1275 (137)	Deep learning	AUC: 0.76-0.82
		NS		
		2 external test sets		
Ye et al. (2021)^52^	US	934 (110)	Deep learning	AUC: 0.90; Acc: 88.9%
		85%/15%		
Zhang et al. (2021)^53^	US	906 (158)	Deep learning	AUC: 0.86
		80%/20%		
Zhang et al. (2021)^36^	DCE-MRI	99 (10)	Deep learning	AUC: 0.89; Acc: 89.0%
		10-fold CV		
Ge et al. (2022)^62^	DM	319 (65)	Radiomics + ML	AUC: 0.81; Acc: 80.6%
		65%/35%		
Du et al. (2022)^54^	US	360 (178)	Radiomics + ML	AUC: 0.98
		70%/30%		
Dominique et al. (2022)^63^	CEM	447 (35)	Deep learning	AUC: 0.91; Acc: 84.7%
		65%/15%/20%		
Boulenger et al. (2023)^55^	US	145 (16)	Deep learning	AUC: 0.86; Acc: 85%
		80%/10%/10%		
Yin et al. (2023)^43^	DCE-MRI, DWI	319 (154)	Deep learning	AUC: 0.94; Acc: 94.0%^a^
		60%/20%/20%		
Yue et al. (2023)^35^	DCE-MRI	516 (72)	Deep learning	AUC: 0.93; Acc: 91.0%
		80%/20%		
Gong et al. (2023)^56^	US, CE-US	120 (16)	Radiomics + ML	AUC: 0.83; Acc: 86.9%
		80%/20%		
Shuangshuang et al. (2023)^65^	CEM	322 (47)	Radiomics + ML	AUC: 0.69-0.87; Acc: 75.0-91.0%
		50%/50%		
		1 external test set		
Zhang et al. (2023)^45^	MP-MRI	477 (74)	Radiomics + ML	AUC: 0.90
		80%/20%		
Huang et al. (2024)^44^	MP-MRI	188 (33)	Radiomics + ML	AUC: 0.86
		70%/30%		

Unless otherwise noted, studies report on TNBC vs. non-TNBC classification performance (cross-validated or in withheld test sets). Data splits indicate the distributions of the dataset into Train/Validation/Test or Train/Test sets during internal development and testing.

Abbreviations: CE-CT = contrast-enhanced computed tomography; CEM = contrast-enhanced mammography; CE-US = contrast-enhanced ultrasound; DCE-MRI = dynamic contrast-enhanced MRI; CE-DCE-MRI = contrast-enhanced DCE-MRI; DM = digital mammography; DWI = diffusion-weighted MRI; FDG-PET/CT = ^18^F-fluorodeoxyglucose positron emission tomography/computed tomography; MP-MRI = multiparametric MRI; SM = synthetic mammography acquired with breast tomosynthesis; US = ultrasound; NS = data splits not specified; PRoRE = prospective-data-collection, retrospective-evaluation; LOOCV = leave-one-out cross-validation; CV = cross-validation; Acc = accuracy; AUC = area under the ROC curve.

a Performance on TNBC vs. fibroadenoma classification.

b No data splits or cross-validation scheme used.

Across breast imaging modalities, radiomics combined with ML classifiers have been the most common AI approach in TNBC classification. Deep learning approaches, leveraging larger sets of ultrasound and MRI examinations (ie, >400 breast cancer patients), have emerged in more recent studies,^35^^,^^50^^,^^52^^,^^53^^,^^63^ and have consistently demonstrated promising classification performances with AUC ≥ 0.74.

### Neoadjuvant treatment response prediction for TNBC

Neoadjuvant treatment (NAT) is intended to reduce the tumour load prior to surgery and may result in pathological complete response (pCR), typically defined as no viable invasive cancer in the breast and lymph nodes.^66^ Achievement of pCR has been widely established as a strong indicator of benefit of NAT and early surrogate for longer recurrence-free survival and better overall survival.^66–68^ However, not all patients respond to NAT, with pCR rates as low as 51% among TNBC patients.^69^^,^^70^ In addition, although important for prognosis, pCR evaluation is assessed in the post-treatment surgical resection specimen, leaving only room for tailoring the treatment post-surgery. At the same time, administering NAT is a process that lasts for several months, has side effects and *de facto* postpones the surgery while the tumour may progress locally and systemically if the patient does not respond. Therefore, there is an urgent need for effective approaches to predict before the start of NAT whether treating a TNBC patient will result in pCR, allowing for personalized changes to treatment plans, including targeted therapies and early discontinuation of ineffective therapies, to maximize the treatment’s benefit-to-risk ratio. As such, multiple research efforts have been devoted to radiologic AI methodologies for the prediction of the sensitivity of TNBC to NAT and have found that AI in the form of radiomics combined with ML models^71–77^ can achieve accurate NAT response prediction in TNBC (Table 2).

**Table 2.** Overview of prior AI work on predicting pathologic complete response (pCR) to neoadjuvant treatment among triple-negative breast cancer (TNBC) patients.

Study (year)	Imaging modality	Treatment	Patients/Lesions *N*, All TNBC (pCR) Data splits Use of External Test Set	AI approach	Predictive performance
Golden et al. (2013)^76^	DCE-MRI	NAC	60 (22)^a^	Radiomics + ML	AUC: 0.68
			90%/10%		
Banerjee et al. (2018)^77^	DCE-MRI	NAC	41 (20)^a^	Radiomics + ML	AUC: 0.74
			90%/10%		
Liu et al. (2019)^71^	DCE-MRI, DWI	NAC	11 (9)^a^	Radiomics + ML	AUC: 0.79-0.84
			NS		
			2 external test sets		
Choudhery et al. (2020)^72^	DCE-MRI	NAC	74 (27)^a^	Radiomics + ML	AUC: 0.73
			90%/10%		
Huang et al. (2021)^107^	CE-CT	NAST	32 (18)^b^	Radiomics + ML	AUC: 0.84; Acc: 0.84
			90%/10%		
Jiang et al. (2021)^81^	US	NAC	592 (35)^a^	Radiomics + deep learning	AUC: 0.93; Acc: 0.84
			60%/40%		
Jimenez et al. (2022)^73^	DCE-MRI	NAST	80 (33)^a^	Radiomics + ML	AUC: 0.71
			80%/20%		
Roy et al. (2022)^108^	FDG-PET	NAC	20 (10)^a^	Radiomics + ML	Acc: 0.72
			90%/10%		
Zhang et al. (2022)^74^	DCE-MRI	NAC	112 (28)^a^	Radiomics + ML	AUC: 0.73
			65%/35%		
Caballo et al. (2022)^75^	DCE-MRI	NAC	72 (19)^b^	Radiomics + ML	AUC: 0.80
			LOOCV		
Zhou et al. (2023)^79^	DCE-MRI, DWI	NAST	162 (79)^b^	Deep learning	AUC: 0.83-0.86^c^
			80%/20%		
			1 external test set		Acc: 74%-76%^c^
Hwang et al. (2023)^80^	SyMRI	NAST	181 (88)^a^	Radiomics + ML	AUC: 0.57
			65%/35%		

Unless otherwise noted, studies report on predictive performance (cross-validated or in withheld test sets) of AI models based on features extracted from baseline (pre-treatment) images. Data splits indicate the distributions of the dataset into Train/Validation/Test or Train/Test sets during internal development and testing.

Abbreviations: CE-CT = contrast-enhanced computed tomography; DCE-MRI = dynamic contrast-enhanced MRI; DWI = diffusion-weighted MRI; FDG-PET = ^18^F-fluorodeoxyglucose positron emission tomography; SyMRI = synthetic MRI; NS = data splits not specified; LOOCV = leave-one-out cross-validation; Acc = accuracy; AUC = area under the ROC curve; NAC = neoadjuvant chemotherapy; NAST = neoadjuvant systemic therapy.

a pCR defined as the absence of residual invasive disease in the breast and lymph nodes.

b pCR defined as the absence of residual invasive and in-situ disease in the breast and lymph nodes.

c Studies where the reported performance is for a combination of features extracted from baseline images and images acquired during neoadjuvant treatment.

Golden et al.^76^ was among the first studies that showed the potential of radiomic tumour features derived from pre-treatment DCE-MRI to predict pCR to NAT among TNBC patients. Using a small cohort of 60 TNBC patients and radiomic features based on grey-level co-occurrence matrices (GLCM), the authors showed that pre-NAT quantitative imaging features were able to predict pCR with AUC = 0.68, as well as residual lymph node metastases with AUC = 0.84 and residual tumour with lymph node metastases with AUC = 0.83. In a follow-up study,^77^ the same group expanded the set of tumour imaging features to a wide range of radiomic features extracted with a multiscale, translation, and rotation-covariant texture analysis framework based on the Riesz wavelets.^78^ Their findings showed that this expanded set of radiomic features could improve their predictive performance by at least 13%, while also providing a more comprehensive imaging phenotype of the TNBC lesions. Since then, the development of radiomic methodologies for pre-treatment NAT response prediction with DCE-MRI in TNBC has been an active area of research.^71–75^ Most studies to date have involved radiomic features related to tumour texture from different temporal phases of DCE-MRI or kinetics for temporal enhancement analysis of the tumour (Table 2). Most recently, to integrate texture and kinetic characteristics from DCE-MRI, Caballo et al.^75^ introduced a novel 4D radiomic approach for the spatio-temporal analysis of pre-NAT DCE-MRI images, which achieved an AUC of 0.80 in the prediction of TNBC patients achieving pCR.

Though most studies have primarily focused on radiomic DCE-MRI features predictive of NAT response, recent work has also explored other breast imaging modalities, such as DWI,^71^^,^^79^ and synthetic MRI.^80^ Moreover, emerging AI developments in this research area include multi-modal deep learning models,^79^^,^^81^ and the integration of tumour and peritumoural areas^82^ as regions of interest. Last, beyond NAT response prediction, recent research efforts also suggest a promising role of AI in predicting response of TNBC patients to immunotherapy, via associations of pre-treatment tumour radiomic features with tumour-infiltrating lymphocytes (TILs),^83^^,^^84^ and PD-L1 expression status,^85^ both significant predictive indicators of TNBC response to immunotherapy treatments.

### Prognosis

Prognostic assessment is a key component of personalized treatment regimens for breast cancer.^86^^,^^87^ In addition to traditional tumour node metastasis (TNM) staging, various clinicopathological tissue-based factors have shown prognostic value in TNBC patients, including the Ki-67 tumour proliferation index,^88^ the presence of TILs,^89^ the neutrophil-to-lymphocyte ratio (NLR),^90^ and upregulation or downregulation of specific miRNAs.^91^ While tissue-based biomarkers are continuously being refined, they are limited by selective tissue sampling which may not fully capture the tumour heterogeneity^92–95^ and additional costs introduced to standard clinical procedures. Therefore, AI applications for routinely collected radiologic images provide a unique means for capturing prognostic characteristics of the entire tumour *in vivo*^9^ at low additional cost in terms of patient engagement and resources. In the last 4 years, various studies have demonstrated a strong prognostic role of AI for TNBC using various clinical endpoints, such as disease-free survival^96–100^ (locoregional or distant cancer recurrence or the development of a new primary contralateral breast cancer) and systemic recurrence^101^^,^^102^ (cancer recurrence in lungs, liver, bones, distant lymph nodes, brain, peritoneum, or adrenal gland) (Table 3).

**Table 3.** Overview of prior work on prognosis for triple-negative breast cancer (TNBC).

Study (year)	Imaging modality	Endpoint (follow-up time)	Patients/Lesions *N*, All TNBC (event) Data splits Use of External Test Set	AI approach	Prognostic performance
Koh et al. (2020)^101^	DCE-MRI	SR (43 ± 16 m)	231 (22)	Radiomics + ML	C-index: 0.85
			80%/20%		
Jiang et al. (2020)^96^	DM	iDFS (range 3-36 m)	200 (17)	Radiomics + ML	C-index: 0.94
			65%/35%		
Kim et al. (2020)^97^	DCE-MRI	DFS (range 5-80 m)	228 (32)	Radiomics + ML	iAUC: 0.70
			75%/25%		
Yu et al. (2021)^98^	US	DFS (range 32-65 m)	324 (93);	Radiomics + ML	C-index: 0.71-0.73
			67%/33%		
			1 external test set		
Xia et al. (2021)^99^	DCE-MRI	DFS (range 1-101 m)	150 (45)	Radiomics + ML	C-index: 0.87
			70%/30%		
Ma et al. (2022)^102^	DCE-MRI	SR (range 1-36 m)	147 (31)	Radiomics + ML	AUC: 0.81
			70%/30%		
Wang et al. (2022)^100^	US	DFS (median 76 m)	562 (68)	Radiomics + ML	AUC: 0.90
			80%/20%		

Unless otherwise noted, studies report on prognostic performance (cross-validated or in withheld test sets) of AI models based on features extracted from baseline (pre-treatment) images. Data splits indicate the distributions of the dataset into Train/Validation/Test or Train/Test sets during internal development and testing.

Abbreviations: DCE-MRI = dynamic contrast-enhanced MRI; DFS = disease-free survival; DM = Digital mammography; iDFS = invasive disease-free survival; SR = systemic recurrence; US = ultrasound; AUC = area under the ROC curve; C-index = concordance index; iAUC = integrated AUC.

Among these studies, Yu et al.^98^ leveraged a total of 486 TNBC patients from 3 different cohorts with the aim to explore the predictive value of a radiomics nomogram from pre-treatment ultrasound in disease-free survival after NAT. The authors used the primary cohort to develop the radiomic nomogram, which was then tested in an internal validation cohort and an external validation cohort. Their findings indicated an effective prognostic performance for the radiomic nomogram, with an internal validation C-index of 0.73 and an external validation C-index of 0.71. In addition, the radiomic nomogram yielded a higher prognostic performance (*P* < 0.05) than a clinicopathological model based on tumour size, axillary lymph node status and Ki-67 index. Similar findings were reported by Ma et al.^102^ based on a total of 147 patients with TNBC, who showed that a radiomic model built based on pre-NAT DCE-MRI features had good performance (AUC = 0.81) in predicting whether patients with TNBC will have a systemic recurrence within 3-year post-neoadjuvant chemotherapy (NAC). Moreover, their radiomic model outperformed (*P* < 0.05) a clinical prognostic model (AUC = 0.75) based on clinical tumour stage and pathological lymph node stage.

## Discussion

The rise and dissemination of AI in radiologic breast imaging is well poised to advance precision oncology, with TNBC already benefiting from promising research results. Key clinical applications include the differential diagnosis of TNBC, early prediction of response to NAT, and prediction of disease recurrence. Radiomics combined with traditional ML classifiers have been the key player in these AI applications for TNBC, with deep learning emerging in more recent research, primarily for differentiating TNBC from other molecular breast cancer subtypes. In terms of radiologic imaging modality, DCE-MRI and ultrasound have been the most commonly used input to predictive and prognostic AI models for TNBC, while few studies have explored the potential of imaging biomarkers extracted from mammography, CT, and PET.

Moving forward, we identify (a) large heterogenous datasets, (b) multi-modal AI approaches, and (c) benchmarking and reproducibility efforts, as 3 key research priorities for AI with the goal of further advancing diagnosis, treatment response predictions and prognostic evaluations for patients with TNBC. Due to the low prevalence of TNBC, sample size has been a major limitation in AI research on TNBC to date, particularly in single-site studies, which has also limited the power to investigate potential effects of race in the predictive and prognostic value of AI-driven radiologic imaging biomarkers. Therefore, large multi-site validation studies that also include racially diverse TNBC cohorts and data from promising clinical trials for TNBC^69^^,^^70^ are essential. Moreover, although prior work consistently supports the predictive and prognostic value of TNBC imaging biomarkers from a single radiologic imaging modality, the synergistic role of biomarkers extracted from different breast imaging modalities remains largely unexplored. Multi-modal AI approaches to TNBC tumour imaging phenotyping, integrating complementary radiologic breast imaging modalities^103^ and possibly also histopathology data,^10^ can result in cross-modal patterns of superior predictive and prognostic performance for TNBC. Last, benchmarking efforts allowing the evaluation of the relative performance of different AI models for TNBC on the same datasets are essential to develop more robust and reproducible imaging biomarkers. Currently, there are various publicly available breast cancer imaging databases.^32^^,^^104^^,^^105^ Moreover, the “Breast Multiparametric MRI for prediction of NAC Response (BMMR2) Challenge” is an important initiative focusing on identifying MRI biomarkers for predicting pCR following NAT for invasive breast cancer, with participation from multiple research teams.^106^ However, public databases and benchmarking efforts with higher representation of TNBC are still needed. In addition to improving reproducibility in AI, such initiatives can significantly enhance the transparency and therefore, the trust, in AI models for TNBC, accelerating their transition into clinical implementation.

In addition, despite promising AI advances, several challenges remain in translating AI-based methodologies into routine clinical practice. One key limitation is the lack of automation in radiomic workflows, which are still highly dependent on manual segmentation and region-of-interest (ROI) selection, making them time-consuming and susceptible to annotator bias. This human involvement not only increases the complexity and time required for analysis but also introduces variability that can affect the reproducibility of results across different clinical settings. Additionally, the multi-step image processing often required for radiomics can create a significant workload, adding to the already complex clinical environment. Another major obstacle is the integration of AI into existing clinical decision support systems (CDSS), where the lack of standardization and interoperability between AI tools and clinical infrastructures presents a considerable barrier. Addressing these challenges by automating image processing steps, mitigating annotator bias, and streamlining AI integration into clinical workflows will be critical to ensuring the real-world applicability of AI tools for TNBC management, and beyond.

In conclusion, data from this review supports the potential of radiologic AI-driven imaging biomarkers to serve as companion tools for guiding the diagnosis and treatment decisions for TNBC. Before clinical adoption of AI for TNBC becomes a reality, various challenges need to be addressed, including IT infrastructure, training of healthcare workforce for AI use, cost-effectiveness, and regulatory approval pathways. Future progress in these domains and new AI developments for TNBC may result in novel paradigm shifts towards diagnosis and treatment decisions for TNBC in the era of personalized medicine.
